# Performance of a dual-hormone closed-loop system versus insulin-only closed-loop system in adolescents with type 1 diabetes. A single-blind, randomized, controlled, crossover trial

**DOI:** 10.3389/fendo.2023.1073388

**Published:** 2023-01-23

**Authors:** Emilie Bundgaard Lindkvist, Christian Laugesen, Asbjørn Thode Reenberg, Tobias Kasper Skov Ritschel, Jannet Svensson, John Bagterp Jørgensen, Kirsten Nørgaard, Ajenthen G. Ranjan

**Affiliations:** ^1^ Copenhagen University Hospital - Steno Diabetes Center Copenhagen, Herlev, Denmark; ^2^ Department of Clinical Medicine, Faculty of Health and Medical Sciences, University of Copenhagen, Copenhagen, Denmark; ^3^ Department of Applied Mathematics and Computer Science, Technical University of Denmark, Kgs. Lyngby, Denmark; ^4^ Department of Pediatrics, Herlev and Gentofte University Hospital, Herlev, Denmark; ^5^ Danish Diabetes Academy, Odense, Denmark

**Keywords:** type 1 diabetes mellitus, adolescents, dual-hormone, advanced hybrid closed-loop, artificial pancreas, non-linear model predictive control, moderate intensity continuous exercise

## Abstract

**Objective:**

To assess the efficacy and safety of a dual-hormone (DH [insulin and glucagon]) closed-loop system compared to a single-hormone (SH [insulin only]) closed-loop system in adolescents with type 1 diabetes.

**Methods:**

This was a 26-hour, two-period, randomized, crossover, inpatient study involving 11 adolescents with type 1 diabetes (nine males [82%], mean ± SD age 14.8 ± 1.4 years, diabetes duration 5.7 ± 2.3 years). Except for the treatment configuration of the DiaCon Artificial Pancreas: DH or SH, experimental visits were identical consisting of: an overnight stay (10:00 pm until 7:30 am), several meals/snacks, and a 45-minute bout of moderate intensity continuous exercise. The primary endpoint was percentage of time spent with sensor glucose values below range (TBR [<3.9 mmol/L]) during closed-loop control over the 26-h period (5:00 pm, day 1 to 7:00 pm, day 2).

**Results:**

Overall, there were no differences between DH and SH for the following glycemic outcomes (median [IQR]): TBR 1.6 [0.0, 2.4] vs. 1.28 [0.16, 3.19]%, p=1.00; time in range (TIR [3.9-10.0 mmol/L]) 68.4 [48.7, 76.8] vs. 75.7 [69.8, 87.1]%, p=0.08; and time above range (TAR [>10.0 mmol/L]) 28.1 [18.1, 49.8] vs. 23.3 [12.3, 27.2]%, p=0.10. Mean ( ± SD) glucose was higher during DH than SH (8.7 ( ± 3.2) vs. 8.1 ( ± 3.0) mmol/L, p<0.001) but coefficient of variation was similar (34.8 ( ± 6.8) vs. 37.3 ( ± 8.6)%, p=0.20). The average amount of rescue carbohydrates was similar between DH and SH (6.8 ( ± 12.3) vs. 9.5 ( ± 15.4) grams/participant/visit, p=0.78). Overnight, TIR was higher, TAR was lower during the SH visit compared to DH. During and after exercise (4:30 pm until 7 pm) the SH configuration produced higher TIR, but similar TAR and TBR compared to the DH configuration.

**Conclusions:**

DH and SH performed similarly in adolescents with type 1 diabetes during a 26-hour inpatient monitoring period involving several metabolic challenges including feeding and exercise. However, during the night and around exercise, the SH configuration outperformed DH.

## 1 Introduction

People with type 1 diabetes (T1D) are advised to aim for near-normal blood glucose levels to reduce the risk of diabetes late complications (1, 2). However, achieving optimal metabolic control is challenging and many fail to meet recommended guidelines – especially adolescents (3).

The most advanced commercially available technology is a single-hormone (SH) closed-loop system, also known as an artificial pancreas (AP). These systems automatically adjust insulin pump delivery based on real-time values from a continuous glucose monitor (CGM). Relative to insulin pump and CGM systems without automated insulin dosing, APs have been shown to improve glucose control (4–6). Despite these technological improvements, adolescents with T1D still frequently experience non-severe hypoglycemia (<3.9 mmol/L) (5, 7). Furthermore, around exercise the risk of hypoglycemia is higher due to increased insulin sensitivity, insulin absorption, glucose uptake in combination with an impaired glucagon secretion (8).

A potential means of reducing the risk of hypoglycemia using closed-loop systems is to add the glucose-elevating hormone glucagon. Such dual-hormone (DH) hybrid closed-loop systems are not currently commercially available but have generated interest in research trials investigating their performance relative to SH systems. A meta-analysis found that both SH and DH closed-loop systems resulted in more time spent in the target glucose range (TIR [3.9-10.0 mmol/L]) compared to non-automated delivery systems. Furthermore, DH was superior to SH in increasing TIR and decreasing time below range (TBR [< 3.9 mmol/L]) (9). Limited data exist comparing DH and SH closed-loop treatments during and after exercise, however, some studies have found DH to minimize TBR in such circumstances (10–13).

Our group has developed the DiaCon AP (14) which can run in two configurations; SH and DH. A previous version of the system was tested among adults with T1D showing improvements in TIR during exercise and a lesser need for hypoglycemic-CHO treatments when using the DH configuration (15). However, the updated system is yet to be tested in an adolescent T1D cohort.

The aim of this study was therefore to test our hypothesis, that the updated DiaCon AP DH configuration would be safe and effective to use in individuals with T1D between 13-17 years old and that it would be *superior* in managing glycemia compared to the DiaCon AP SH configuration.

## 2 Materials and methods

### 2.1 Methods

This was a randomized, single-blind, two-period, crossover study in adolescents with T1D recruited from Herlev and Gentofte Hospital Pediatric Department Outpatient Clinics and the Steno Diabetes Center Copenhagen. Enrollment was conducted from September 1^st^, 2021 until March 7^th^, 2022. All study participants’ parents or legal guardians provided written informed consent and participants ≥15-years provided written, informed assent before participation. The study was approved by the Regional Committee in Health Research Ethics (H-21000207), the Danish Data Protection Agency (P-2021-326), and the Danish Medicines Agency (2020-005836-31). The trial was registered with ClinicalTrials.gov (NCT04949867).

Participants were included if they were: 13-17 years old; diagnosed with T1D for ≥two years; used an insulin pump for ≥one year; used a real-time or intermittently scanned CGM, had an HbA1c ≤75 mmol/mol; and used carbohydrate counting as well as the pump bolus calculator for all meals. Main exclusion criteria were known allergy to glucagon or lactose, use of diabetes medication other than insulin, and hypoglycemia unawareness.

### 2.2 Study device and drugs

We used our DiaCon system consisting of two Dana Diabecare RS insulin pumps (one for insulin and one for glucagon/saline), a Dexcom G6 sensor (Dexcom, San Diego, CA) and a smartphone (Samsung Galaxy A5 2017 Android phone) containing the DiaCon algorithm (14) to adjust the pump deliveries based on the CGM values. One pump was filled with insulin aspart (Fiasp^®^, Novo Nordisk, Bagsværd, Denmark) and the other with either glucagon (GlucaGen^®^, Novo Nordisk, Bagsværd, Denmark) or isotonic saline (sodium chloride 9 mg/dL). The glucagon pump was refilled with fresh glucagon every 22 hours after the first pump filling. The individual parameter estimates for the insulin algorithm were set up using insulin pump, CGM and carbohydrate intake (CHO) data from each participant. The algorithm computed the insulin and glucagon administration based on predictions obtained with a mathematical model of the blood glucose response to CHO, insulin, and glucagon, i.e., based on nonlinear model predictive control (NMPC). Safety constraints on bolus and basal insulin were based on participants’ insulin pump settings and the glucagon algorithm was constrained to deliver maximally 300 µg glucagon over a two hour period (14). Meals and exercise were announced to the DiaCon AP. The NMPC utilized insulin-carbohydrate-ratio and the announced meal carbohydrates when dosing meal boluses. Exercise mode increased target glucose from 6 to 7 mmol/L and the limit for when glucagon could be administered was increased from 4.5 to 7.0 mmol/L. If SG was ≤7.0 mmol/L at exercise announcement 100 µg glucagon was administered (14).

### 2.3 Study design

**Figure 1:**
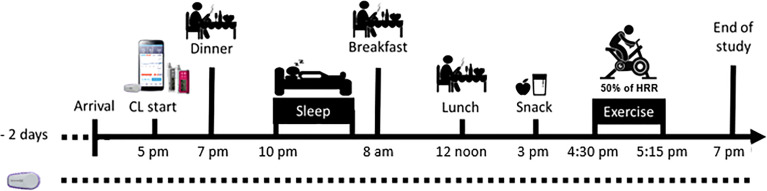
Schematic overview of the study days.

Participants went through a screening visit and two 26-h in-clinic visits with a wash-out period of at least three days. During each in-clinic visit, participants wore the DiaCon system set-up to run in either the DH or SH configuration depending on the randomization order. Except for the DH and SH configurations, the study visits were identical (**Figure 1**).

At the screening visit, participants’ medical history (i.e., allergies, medications, other illnesses, diabetes complications) as well as results of blood and urine analyses were reviewed. A clinical examination was performed to assess height, weight, and blood pressure. Finally, 14 days of insulin pump and CGM data were downloaded to register the mean values for basal rate delivery, insulin sensitivity factor, insulin-carbohydrate-ratio, CHO, TBR, TIR, time above range (TAR [>10.0 mmol/L]) and mean glucose.

**Figure 2:**
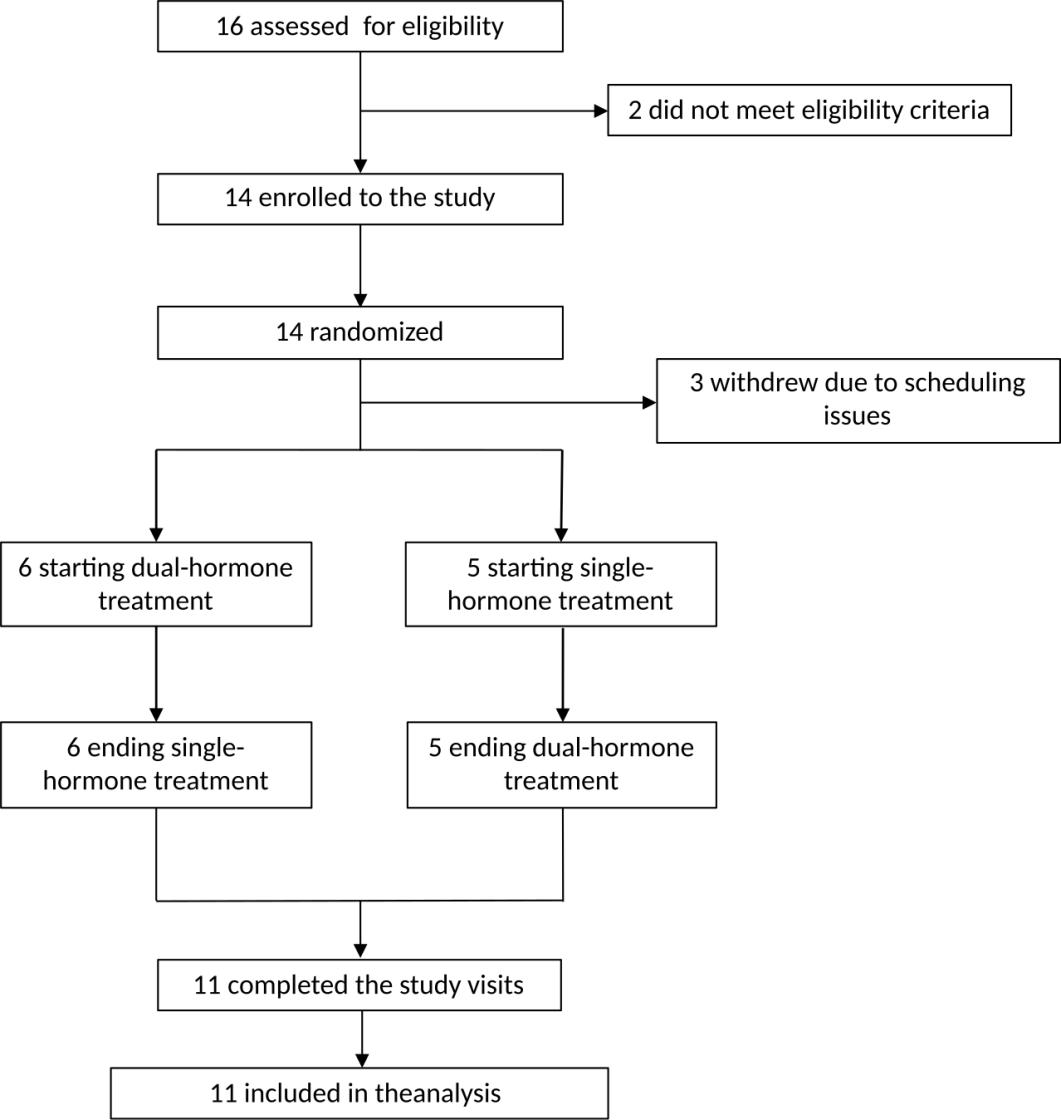
Flowchart of study.

Two days prior to each in-clinic visit, participants inserted the Dexcom G6 sensor which linked to the Dexcom receiver. On visit days, participants arrived at the research facility at 4 pm following a three hour fast. Upon arrival, the Dexcom sensor was linked to the study equipment, an intravenous canula was inserted for blood sampling, and participants were fitted with an activity tracker (ActiGraph GT9X Link, Pensacola, FL). Intervention with one of the two closed-loop configurations was initiated at 5:00 pm and continued for the following 26-h (**Figure 2**). Meals (7:00 pm, 8:00 am and 12:00 noon) and snacks (3:00 pm) were served throughout the inpatient period and their CHO contents were based on the participants’ average daily CHO intake entered in their own pumps evaluated over a seven-day period. Participants eating <100 g daily received 30 g CHO per meal, 100-150 g daily received 50 g CHO per meal, 150-200 g daily received 60 g CHO per meal, and >200 g per day received 70 g CHO per meal. The snack consisted of 1/3 of the CHO given per meal, and the dinner consisted of 1.5 times the CHO content of the other regular meals. The dinner was from McDonalds and the CHO content was determined using their nutrition calculator by the study personnel (16), who also prepared the remaining meals incl. snack. The CHO contents of all meals were blinded from participants, who made estimations which were used as the value entered into the DiaCon AP at the start of each meal. Though meals were kept identical between visits, participants’ estimations could differ.

Participants were instructed to be in bed, and sleep, if possible, from 10:00 pm to 7:30 am.

After resting during the day, at 4:30 pm on day two, participants performed a 45-minute bout of moderate intensity continuous exercise at an intensity equivalent to ~ 50% of their heart rate (HR) reserve (17). Participants were fitted with a chest strap telemetry monitor that linked to the ActiGraph and the stationary bike. Exercise duration was announced to the DiaCon AP upon initiation of exercise. During the entirety of the study period, participants were asked to stay around the research facility, not to exercise and eat, and to only be away from the research room within the 30-minute window between plasma samples.

Venous blood samples were drawn every 30 minutes during the day, every 60 minutes during sleep (10:00 pm until 7:30 am) and every five to ten minutes during and immediately after exercise (4:30 pm until 5:30 pm). Plasma glucose (PG) was measured *via* the YSI 2300 STAT Plus Analyzer (YSI Life Sciences, Yellow Springs, OH).

If PG dropped <3.0 mmol/L at any time during the intervention, 15 grams of oral rescue carbohydrate (dextrose) tablets were provided to the participants, and plasma sampling was performed every five minutes. The treatment was repeated every 15 minutes until PG was >3.9 mmol/L. If PG was >12 mmol/L for more than two hours or >14 mmol/L (not in relation to a meal), blood ketones were measured in 15 minutely intervals and the study devices were checked for issues. After an hour, if blood ketones were ≥0.6 mmol/L and PG remained >14 mmol/L, insulin was administered with an injection pen based on the participants’ insulin-sensitivity factor aiming for PG of 7.0 mmol/L.

During each visit, participants scored side effects using a visual analog scale (VAS; 0-100) at seven specified timepoints (day 1: 5:00 pm and 7:00 pm and day 2: 8:00 am, 12:00 noon, 3:00 pm, 4:30 pm and 7:00 pm). Clinically significant side effects were defined as a VAS score ≥ 15 (18–20).

### 2.4 Outcomes

The primary outcome was percentage of TBR_SG_ during the 26-h intervention period. Secondary outcomes were: percentage of TBR_PG_, percentage of TIR_SG&PG_ and TAR_SG&PG_, mean SG and PG, coefficient of variation (CV) and number of rescue CHO interventions. Study outcomes were also reviewed separately overnight (10:00 pm to 7:30 am) as well as during and after exercise (4:30 pm to 7:00 pm).

### 2.5 Statistical analysis

To be able to detect a difference in percentage of time with SG <3.9 mmol/L of 2.3%-points (approximately 30 minutes) with 90% power, a 5% significance level, and a presumed 3.0%-points standard deviation (10), it was established that 20 participants were to be included in the study (2-sided test). Categorical variables were reported as frequencies (percentage), whereas continuous variables were reported as mean (SD) or median (interquartile range [IQR]). Continuous data was assessed for normality using Shapiro-Wilk test. For normally distributed variables, paired student’s t-test was used to conduct pairwise comparisons between the two groups. For skewedly distributed variables despite log-transformation, the non-parametric Wilcoxon signed-rank test was used. Missing glucose data were estimated using linear interpolation. We used McNemar’s test to assess the significance of the difference in the incidence of level 2 hypoglycemia between the two study arms. Analyses were performed on an intention-to-treat basis. Statistical analyses were performed using RStudio version 1.4.

## 3 Results

As per protocol, we performed an interim analysis to assess efficiency of the DiaCon algorithm, where we found DH to be inferior to SH for TIR and TAR and to be non-superior for TBR, therefore the inclusion was truncated. At that time, 16 had been screened and 14 were included and enrolled in the study. Two adolescents were not eligible due to hypoglycemia unawareness. Before initiation of the first study visit, three participants withdrew due to scheduling issues [e.g., school absence and lack of time (**Figure 2**)]. Thus, 11 participants completed both visits (**Table 1**), and no differences were observed between completers and non-completers on age, sex, BMI, HbA1c, diabetes duration or daily insulin dose.

**Table 1:** Baseline characteristics of the 11 participants who completed both study visits.

Baseline characteristics	Mean ( ± SD) or median [IQR]
Sex (males [%])	9 (82%)
Age (years)	14.8 ( ± 1.47)
HbA1c (mmol/mol)	54.6 ( ± 9.20)
BMI (kg/m^2^)	21.4 ( ± 2.42)
Diabetes duration (years)	5.73 ( ± 2.45)
Total daily insulin (U/kg)	0.94 ( ± 0.26)
Time below range (%)	3.0 [1.5, 6.5]
Time in range (%)	54.0 [46.0, 73.0]
Time above range (%)	43.0 [22.5, 52.0]

Age, HbA1c, BMI, diabetes duration and total daily insulin are expressed as mean ( ± SD). Time below (<3.9 mmol/L), in (3.9-10.0 mmol/L) and above range (>10.0 mmol/L) are expressed as median [interquartile range].Sex is presented as absolute number and percentage.

### 3.1 Entire study period

#### 3.1.1 Glycemic metrics

For the 26-h study period we found no differences in TBR_SG_, TIR_SG_, or TAR_SG_ between the two study arms (**Table 2**). The mean ( ± SD) SG was higher during the DH compared to the SH study arm (8.7 ( ± 3.0) mmol/L vs. 8.1 ( ± 3.0) mmol/L, p<0.001), with no difference in CV. Similarly, no differences were found in PG-derived measures (**Table 2**), except for TAR_PG_ (33.2 [16.1, 40.7] vs. 11.5 [3.83, 23.0]%, p=0.02) and mean_PG_ (8.84 ( ± 2.83) vs. 7.51 ( ± 2.98), p=0.03) both of which were higher during DH.

**Table 2:** Sensor and plasma glucose values during entire study, overnight and exercise and post-exercise period.

	Sensor Glucose Measures			Plasma Glucose Measures		
	Dual-Hormone (n=11)	Single-Hormone (n=11)	*P-value*	Dual-Hormone (n=11)	Single-Hormone (n=11)	*P-value*
Entire study period (5:00 pm, day 1 – 7:00 pm, day 2)						
Starting glucose for period (mmol/L)	7.33 ( ± 1.90)	9.35 ( ± 6.61)	0.64^w^	6.61 ( ± 1.71)	9.14 ( ± 4.90)	0.21
Time below range (%)	1.60 [0, 2.4]	1.28 [0.16, 3.19]	1.00^w^	0.958 [0, 3.83]	2.56 [0.479, 8.47]	0.26^w^
Time in range (%)	68.4 [48.7, 76.8]	75.7 [69.8, 87.1]	0.09	66.8 [56.9, 78.9]	79.6 [75.2, 87.4]	0.06
Time above range (%)	28.1 [18.1, 49.8]	23.3 [12.3, 27.2]	0.10	33.2 [16.1, 40.7]	11.5 [3.83, 23.0]	**0.02**
Mean glucose (mmol/L)	8.7 ( ± 3.02)	8.1 ( ± 3.0)	**<0.001**	8.84 ( ± 2.83)	7.51 ( ± 2.98)	**0.03**
Coefficient of variation (%)	34.8 ( ± 6.8)	37.3 ( ± 8.6)	0.20	33.59 ( ± 8.17)	39.63 ( ± 8.72)	0.17
Overnight period (10:00 pm – 7:30 am)						
Starting glucose for period (mmol/L)	10.3 ( ± 3.55)	9.34 ( ± 3.66)	0.54	9.66 ( ± 3.53)	8.22 ( ± 3.71)	0.24
Time below range (%)	0 [0, 0]	0 [0, 3.48]	0.10^w^	0 [0, 0]	0 [0, 5.22]	0.36^w^
Time in range (%)	73.0 [53.9, 87.4]	96.5 [84.3, 100]	***0.02*** ^w^	67.8 [60.4, 89.6]	89.6 [82.6, 100]	0.07^w^
Time above range (%)	27.0 [12.6, 42.6]	0 [0, 10.0]	***0.02*** ^w^	32.2 [5.22, 37.8]	0 [0, 5.22]	**0.02** ^w^
Mean glucose (mmol/L)	8.49 ( ± 2.97)	7.25 ( ± 2.25)	**0.04**	8.34 ( ± 2.70)	6.88 ( ± 2.38)	**0.01**
Coefficient of variation (%)	35.0 ( ± 10.8)	31.0 ( ± 8.9)	0.39	32.34 ( ± 11.62)	34.66 ( ± 9.78)	0.69
Exercise and post-exercise period (4:30 pm – 7:00 pm)						
Starting glucose for period (mmol/L)	10.1 ( ± 3.15)	9.31 ( ± 2.30)	0.35	9.40 ( ± 2.92)	7.71 ( ± 2.25)	0.14
Time below range (%)	0 [0, 0]	0 [0, 0]	0.42^w^	0 [0, 4.84]	0 [0, 14.5]	0.78^w^
Time in range (%)	64.5 [50.0, 91.9]	83.9 [80.6, 100]	***0.02*** ^w^	67.7 [32.3, 99.4]	93.5 [82.3, 100]	0.10^w^
Time above range (%)	22.6 [0, 37.1]	12.9 [0, 17.7]	0.06^w^	1.18 [0, 58.1]	0 [0, 3.23]	0.09^w^
Mean glucose (mmol/L)	7.92 ( ± 2.92)	6.81 ( ± 1.91)	0.13	7.91 ( ± 2.70)	6.06 ( ± 1.76)	**0.05**
Coefficient of variation (%)	37.0 ( ± 9.6)	28.2 ( ± 7.7)	0.65	34.16 ( ± 11.51)	29.10 ( ± 7.49)	0.49
Change_during-exercise_ (mmol/L)	-2.5 ( ± 1.6)	-3.2 ( ± 1.0)	0.14	-2.5 ( ± 1.8)	-2.7 ( ± 1.7)	0.73
Heart Rate Telemetry during exercise (4:30 pm – 5:15 pm)						
HR target_during-exercise_ (BPM)	133 ( ± 4.6)	132 ( ± 4.8)	0.33			
HR target accuracy_during-exercise_ (%)	94.6 ( ± 5.7)	94.2 ( ± 4.6)	0.52			

Time below (<3.9 mmol/L), in (3.9-10.0 mmol/L) and above range (>10.0 mmol/L) are reported as median [interquartile range], starting glucose, mean glucose and coefficient of variation are reported as mean ( ± SD) and level 2 hypoglycemia is presented as events/participant. Time in range 3.9-10.0 mmol/L, time below range <3.9 mmol/L and time above range >10.0 mmol/L. ^w^non-parametric test. For level 2 hypoglycemic events McNemars test was performed. HR, Heart Rate; BPM, Beats per minute. Measures notated with _during-exercise_ depicts the 45-minutes bout of exercise time period.

P-values marked with w have been analysed using non-parametric test. Otherwise, paired t-tests have been applied. P-values < 0.05 were considered statistically significant (marked bold).

During DH, six events of SG-derived level 2 hypoglycemia (<3.0 mmol/L) were registered in three participants compared to two events in two participants during SH (p=0.65). In contrast, four episodes of PG-derived level 2 hypoglycemia were registered in three participants during DH compared to five episodes in four participants during SH (p=0.71). Of those, two of the events were prolonged (>20 minutes) during DH compared to one during SH.

**Figure 3:**
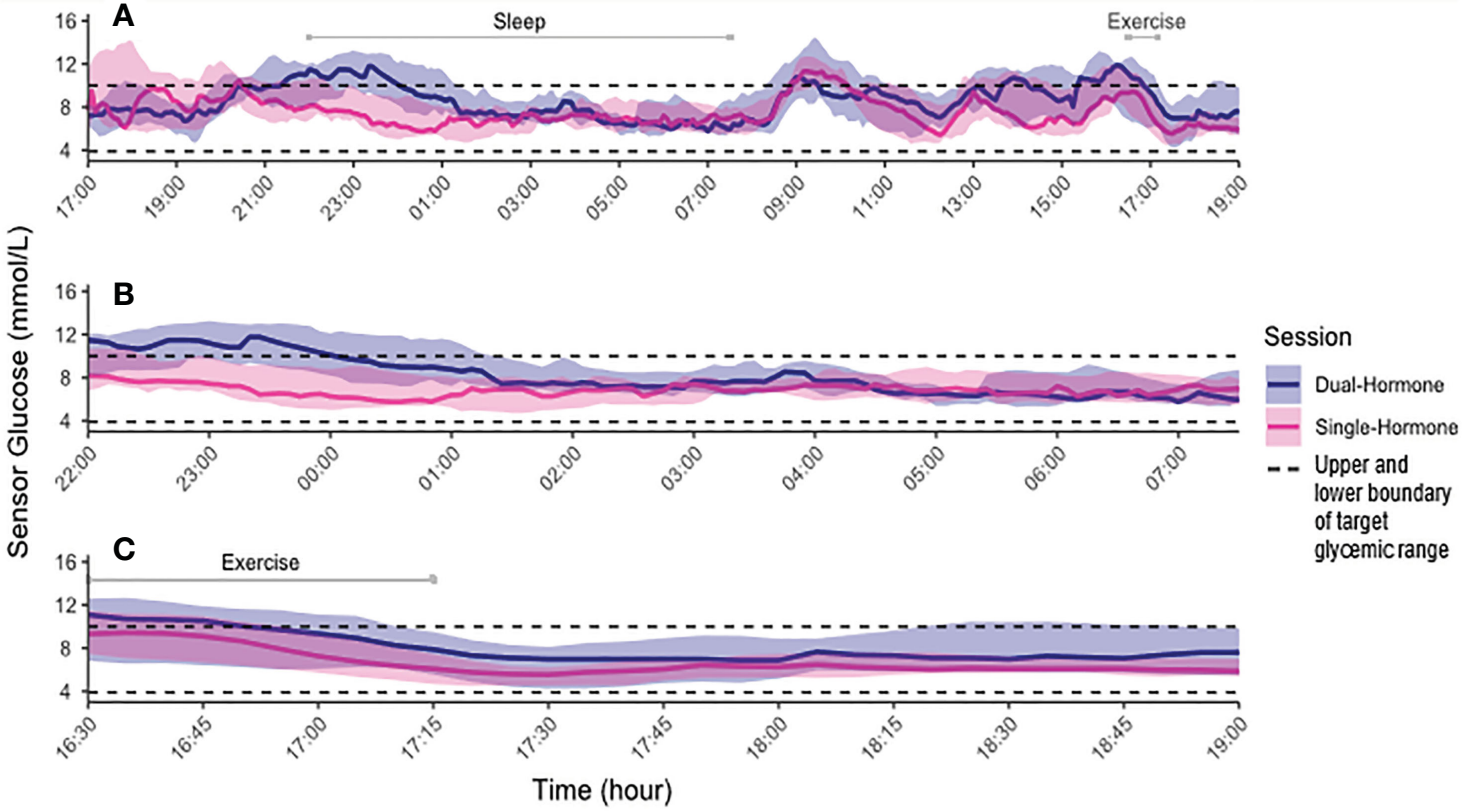
Median (interquartile range) sensor glucose values for Dual-hormone (blue) and Single-Hormone (pink) configuration. **(A)** Entire period. **(B)** Overnight period (10:00 pm-7:30 am). **(C)** Exercise and post-exercise period. Black dotted lines mark lower and upper boundary of target glycemic range of 3.9 to 10.0 mmol/L, respectively.

**Figure 3** shows the SG profile during the total study period, overnight and the period during and after exercise. Individual SG profiles are provided in the **Supplemental Material, Figure 1S**.

#### 3.1.2 Insulin and glucagon

There were no differences in insulin delivery, either total insulin delivery or average basal rate, between the DH and SH study arms (**Table 3**, **Supplemental Material Figures 2-5S**). For the entire study period, all received glucagon with a median [IQR] administration of 549 [229, 1034] µg (**Figures 6-7S**).

**Table 3:** Study medication administration. Insulin measures are reported as mean (SD) and glucagon as median [IQR].

Insulin and glucagon delivery	Dual-Hormone (N=11)	Single-Hormone (N=11)	*P-value*
Entire study period (5:00 pm, day 1 – 7:00 pm, day 2)			
Total insulin delivery (U/24h)	54.8 (19.0)	54.6 (15.4)	0.95
Basal insulin delivery (U/h)	1.03 (0.36)	1.01 (0.30)	0.77^w^
Bolus insulin delivery (U/24h)	29.9 (12.4)	30.3 (9.6)	0.92
Glucagon delivery (µg/26-h)	549 [229, 1034]	-	-
Overnight period (10:00 pm – 7:30 am)			
Total insulin delivery (U/night)	12.94 (4.14)	10.51 (3.08)	**0.01**
Basal insulin delivery (U/h)	1.09 (0.37)	1.05 (0.34)	0.15^w^
Bolus insulin delivery (U/night)	2.55 (2.50)	0.5 (1.13)	**0.04^w^**
Glucagon delivery (µg/night)	298 [0, 462]	-	-
Exercise and post-exercise period (4:30 pm – 7:00 pm)			
Total insulin delivery (U/exercise)	2.24 (1.07)	2.19 (0.98)	0.88
Basal insulin delivery (U/h)	0.80 (0.38)	0.87 (0.40)	0.51
Bolus insulin delivery (U/exercise)	0.25 (0.47)	0.01 (0.03)	0.14^w^
Glucagon delivery (µg/period)	1 [0, 275]	-	-

P-values marked with w have been analysed using non-parametric test. Otherwise, paired t-tests have been applied. P-values < 0.05 were considered statistically significant (marked bold).

#### 3.1.3 Carbohydrates (rescue interventions and meals)

The mean amount of rescue CHO provided was similar between the two arms (DH: 6.8 ( ± 12.3) grams/participant/visit vs. SH: 9.5 ( ± 15.4) grams/participant/visit, p=0.78).

The median [IQR] amount of CHO provided for each meal was 72 [70, 94] g for dinner, 60 [55, 70] g for breakfast, 60 [55, 70]g for lunch and 21 [19, 24]g for snacks, and we found no difference in the accuracy (%) of the participants’ CHO estimations between the two study arms.

### 3.2 Overnight period

During the overnight period, the SH configuration outperformed the DH with more TIR_SG_ (96.5 [84.3, 100] vs. 73.0 [53.9, 87.4]%, p=0.02), less TAR_SG_ (0 [0.0, 10.0] vs. 27.0 [12.6, 42.6]%, p=0.02) and lower mean SG (**Table 2**). There were no differences in TBR_SG_ or CV. TAR_PG_ was higher during DH than during SH, with no differences in TIR_PG_ or TBR_PG_ (**Table 2**). The overnight amount of total insulin delivered was lower during SH than during DH (**Table 3**). Seven participants received glucagon overnight by the DH system (549 [409, 599] µg). Data for the entire study population is depicted in **Table 3**.

Overnight we found one and zero SG-derived hypoglycemic episodes, respectively, in the DH and SH arm. We did, however, not provide any rescue glucose interventions as PG was never <3.0 mmol/L in either arm.

### 3.3 Exercise period

For the exercise and post-exercise phases, the SH system performed superiorly to DH with more TIR_SG_ (83.9 [80.6, 100.0] vs. 64.5 [50.0, 91.9]%, p=0.02). There were no differences in TAR_SG_, TBR_SG_, mean SG, CV or in PG (**Table 2**). For this period, there was no difference in amount of insulin delivered. A total of five participants received glucagon during and after exercise (300 [250, 300] µg) (**Table 3**).

One participant received rescue CHO following exercise in the DH arm, and two participants received one and two rescue CHO interventions, respectively, in the SH arm. The participant needing two rescue interventions around exercise experienced level 2 hypoglycemia during exercise, and the exercise bout was therefore cut short. None of the DH exercise sessions were interrupted.

The exercise starting SG and drop in SG during exercise (4:30 to 5:15 pm) were comparable between the two study visits (**Table 2**). Furthermore, we found no difference in the estimated HR target during exercise or in the accuracy (%) of which participants reached their target between the two visits (**Table 2**).

### 3.4 Side effects

No severe adverse events were observed. Three participants (27%) reported a clinically significant measure of nausea during the DH arm compared with none in the SH arm. Headache was reported by four (36%) in both the SH and the DH arm. Stomach-ache was reported by two (18%) in the DH arm and one (9%) in the SH arm. None experienced vomiting. All reported side effects were mild in severity and, besides from hypoglycemic- and hyperglycemic episodes, required no intervention.

### 3.5 Technological issues

During the study, there were a few technological issues. One participant (during the DH visit) experienced prolonged pressure induced sensor attenuation causing the sensor to register level 2 hypoglycemia, while PG was well within range. This caused wrongful glucagon administration and subsequent hyperglycemia. During two DH study visits, glucagon occluded the pump which required a change of infusion set. During both study arms, the phone lost connection to the sensor. However, all connection issues were automatically reestablished without intervention from the study personnel. These phone-sensor connection issues only triggered an alarm if they lasted more than 15 minutes, thus shorter connection loses may have been present, without the study personnel being aware. Once during a DH study visit, there was a disconnection between the phone and the insulin pumps which was resolved by restarting the entire system.

## 4 Discussion

In this 26-h inpatient study, we compared the performance of the DiaCon DH and SH configurations in adolescents with type 1 diabetes. For the entire study period, we found no differences in TBR_SG/PG_, TIR_SG/PG_,TAR_SG/PG_, or in the amount of rescue glucose needed. However, despite equivalency in TBR_SG_ during the overnight period and around exercise, SH achieved more TIR_SG_ and less TAR_SG_ compared to DH.

The DiaCon AP has previously been tested in adults with type 1 diabetes (15), showing that DH was superior to SH in handling hypoglycemia. These findings were in line with two systematic reviews and meta-analyses performed in 2018, finding that DH was superior to SH for TBR and TIR (4, 9). Still, only two studies have performed head-to-head comparison of DH and SH in adolescents with type 1 diabetes (21, 22). In line with our findings, the first study showed no significant differences in TIR, TAR or TBR between SH and DH for the adolescents during a 24-h observation period (21). Further, they found a tendency for SH to achieve higher TIR than DH during the overnight period (11:00 pm to 8:00 am), but it did not reach statistical significance. The other study was an overnight study showing that the addition of glucagon significantly improved TIR and reduced the time in level 1 hypoglycemia, but had similar time in level 2 hypoglycemia when compared with SH (22). Both studies included a third study arm with usual care, and generally both SH and DH achieved better glycemic control compared to this arm. Recently, a pooled analysis was performed for nocturnal control in children and adolescents using SH and DH. They found superiority in favor of the DH configuration for TIR, level 1 hypoglycemia and level 2 hyperglycemia (23).

Even though our SH configuration managed to ensure good glycemic control comparable to that of the commercially available APs with a TIR_SG_ of 75% for the whole study period, 83% during exercise and 95% overnight, hypoglycemia still posed a challenge (4, 5). Indeed, seven (64%) participants had at least one hypoglycemic event, whilst six experienced recurrent events. This issue was not resolved by addition of glucagon in our DH configuration. The reason may be attributed to the different conditions for parameter estimations in the DiaCon AP rather than glucagon *per se*. While the insulin algorithm was individualized and parameter estimates were based on individual insulin pump and CGM data, the parameter estimates for the glucagon algorithm were generic and similar for all participants. The uncertainty of the individual glucose response to glucagon (median[IQR] glucagon administration 549 [229, 1034] µg), therefore, produced equally uncertain glucose predictions for the system, resulting in less correct insulin dosing in the DH configuration. In this study, the glucagon sensitivity was not adaptive, and we did not have the data to estimate individual glucagon parameters. Whether addition of these features would have improved the DH study results remain uncertain. The DiaCon AP was further challenged by known technical issues, i.e., glucagon pump occlusions and pressure induced sensor attenuation, both of which unfortunately affected the DiaCon AP predictions and hence, it’s performance during the DH study days. Glucagon occlusions happen quite commonly when using native glucagon due to rapid fibrillation after reconstitution (12, 24–26), and although we tried to avoid them by changing glucagon every 22 hours, they still occurred. Future algorithms would therefore benefit immensely from being able to detect occlusions and pressure induced sensor attenuations to avoid miscommunication between the systems’ predictions and the actual CGM data and hormonal delivery. Still, even with improved occlusion detection in the algorithms, a stable formulation of glucagon is required before it is feasible to be used in a real-world setting. New, soluble formulations have been developed (Dasiglucagon^®^, Baqsimi^™^ and Gvoke^®^/XeriSol^®^), but are still only approved for treatment of severe hypoglycemia. Recently published and ongoing clinical trials have shown promising results in using soluble glucagon for treatment of non-severe hypoglycemia, regardless of whether glucagon has been delivered through automated pumps or *via* pen-injections (13, 27, 28).

In the overnight period participants were administered higher doses of bolus insulin during DH compared to SH (2.55 [ ± 2.50] vs. 0.5 [ ± 1.13] IE). This was most likely due to a couple of different factors. During the DH arm some participants were administered glucagon right before dinner announcement, resulting in inadequate meal bolus insulin, and subsequent hyperglycemia. Due to restrictions in the DiaCon AP around meals this led to insulin corrections being administered in the night hours (14). Another possible explanation could be the occurrence of pressure induced sensor augmentations. These caused wrongful glucagon administration and hyperglycemia, which was followed by increased insulin supply when the augmentation was resolved. Furthermore, a few participants experienced oscillating sensor glucose levels, as they were overcorrected and entered a glucagon-insulin oscillating cycle. With the result from the SH arm in mind (TIR 96.5%) it is fair to speculate whether a specific night setting should be developed, making the glucagon algorithm during the night less aggressive, but as our study was rather small and the glucagon algorithm was faced with several challenges, we still think it is too unsafe to conclude (10).

Initially, we hypothesized that the addition of glucagon could have counteracted the increased risk for hypoglycemia during physical activity (8). However, we found that SH was superior to DH with more TIR_SG_, and no difference in TAR_SG_ or TBR_SG/PG_ during and after exercise. We speculate whether the higher starting glucose level before exercise for DH (10.1 [ ± 3.15] vs. 9.31 [ ± 2.30], p=0.35), though not statistically significant, may have reduced the need for glucagon around exercise – causing a systematic error when interpreting the glucose data that are in favor for SH. In fact, only five participants received glucagon in relation to exercise. Among four adult head-to-head studies, DH was found to be superior to SH in avoiding hypoglycemia during exercise, whereas only one of the studies also included adolescents (11–13, 15). In the combined adult and adolescent study, no differences were found between the two hormonal configurations (21). Unfortunately, no subgroup analysis – adult versus adolescents - for the exercise period was reported. Our study period ended 1 hour and 45 minutes after the exercise bout, and we were therefore only able to investigate a small window of the post-exercise period. Previous studies, however, have shown that SH systems could sufficiently keep glucose in range during the post-exercise period (21, 29). This could indicate that glucagon may be especially beneficial during aerobic exercise, where blood glucose changes rapidly and insulin reduction or suspension is inadequate, but requirement may waiver in the post-exercise period when the pronounced acute glycemic declines induced by exercise have passed (10).

The side effects experienced in this study were mild and self-limiting, but the adolescents reported more clinically significant events of nausea during DH than during SH (27% vs. 0%). The other side effects were equally distributed across both study visits, as reported by other study groups (26). A new outpatient study investigating dasiglucagon pen treatment for non-severe hypoglycemia found a higher occurrence of mild nausea with glucagon treatment, but the participants would still incorporate it into their regular diabetes treatment if possible (30). Taken collectively nausea remains a limitation of DH use and finding ways to resolve such should be a major consideration in future developments.

The current study had some limitations. Firstly, a small sample size makes the generalizability of the study results difficult. Furthermore, the current DiaCon AP set-up was too comprehensive for commercial pump use. Simpler set-up versions of the DiaCon AP, should be developed. The use of native glucagon limited the possibility to be used in real-world settings, but the current data show that soluble glucagon has a similar response. Furthermore, we conducted our study on adolescents of 13-17-years-old. This age group is different from all other due to the considerable hormonal and physiological changes over time. Therefore, it is important that studies with DH and SH APs also include adolescents before concluding whether it is beneficial in this age group. We chose a McDonalds meal as dinner, which is not typically classified as a balanced and healthy meal, however, we decided to do so in order to test our configurations maximally during the limited study time. Meals with a high carbohydrate and fat content are usually more difficultly handled for people with type 1 diabetes and testing our system in such a situation was therefore important. Furthermore, McDonalds is known worldwide making the approach universally applicable. Our participants were overall better regulated during the study days than in their everyday lives, though, we did not have a usual care control arm to test whether the superior glycemic control achieved by the DiaCon AP would persist an inpatient setting. This was the first 26-h inpatient study, during which the DH configuration was tested during multiple metabolic challenges.

## 5 Conclusion

To conclude, the two configurations performed equally well; but during night and exercise, SH achieved better glucose control than DH with equal amount of rescue glucose needed.

Whether it is justifiable to add glucagon to the AP systems remains unanswered. Our SH configuration managed to yield good glycemic control, but hypoglycemia still posed a challenge which neither of our configurations managed to overcome. However, as we become more familiar with the use of glucagon and with more stable glucagon formulations readily available, improved DH algorithms can potentially be a key player in resolving this.

## Data availability statement

The original contributions presented in the study are included in the article/**Supplementary Material**. Further inquiries can be directed to the corresponding authors.

## Ethics statement

The study was approved by the Regional Committee in Health Research Ethics (H-21000207), the Danish Data Protection Agency (P-2021-326), and the Danish Medicines Agency (2020-005836-31). Written informed consent to participate in this study was provided by the participants’ legal guardian/next of kin.

## Author contributions

KN, JS, CL, ATR, JJ, and AGR contributed to conceive and design the study. EL, ATR, CL, AGR, and TR conducted the clinical trial. EL performed data analysis and wrote the manuscript. AGR, KN, JS, and CL supervised. All authors read and approved the final manuscript. All authors contributed to the article and approved the submitted version.

## Supplementary material

Figure 1S: Individual SG measures for participant during the entire study period for dual-hormone (blue) and single-hormone (pink). Black dotted lines mark the lower and upper boundary of target glycemic range of 3.9 mmol/L to 10.0 mmol/L, respectively.

Figure 2S: Mean (±SD) basal insulin (IE) delivered during the entire study period for dual-hormone (blue) and single-hormone (pink). Sleep and exercise periods marked in the figure.

Figure 3S: Mean (±SD) total insulin (IE) delivered during the entire study period for dual-hormone (blue) and single-hormone (pink). Sleep and exercise periods marked in the figure.

Figure 4S: Mean (±SD) bolus insulin (IE) delivered during the entire study period for dual-hormone (blue) and single-hormone (pink). Sleep and exercise periods marked in the figure.

Figure 5S: Individual insulin delivery for each participant during the entire study period for dual-hormone (blue) and single-hormone (pink).

Figure 6S: Mean glucagon delivery during the entire dual-hormone study period. Sleep and exercise periods marked in the figure.

Figure 7S: Individual glucagon delivery for each participant during the entire dual-hormone study period.
