# Supplementary figures and images for: Performance of a dual-hormone closed-loop system versus insulin-only closed-loop system in adolescents with type 1 diabetes. A single-blind, randomized, controlled, crossover trial
Source: Front Endocrinol (Lausanne). 2023 Jan 23;14:1073388. doi: 10.3389/fendo.2023.1073388 (PMC9899880; doi:10.3389/fendo.2023.1073388)

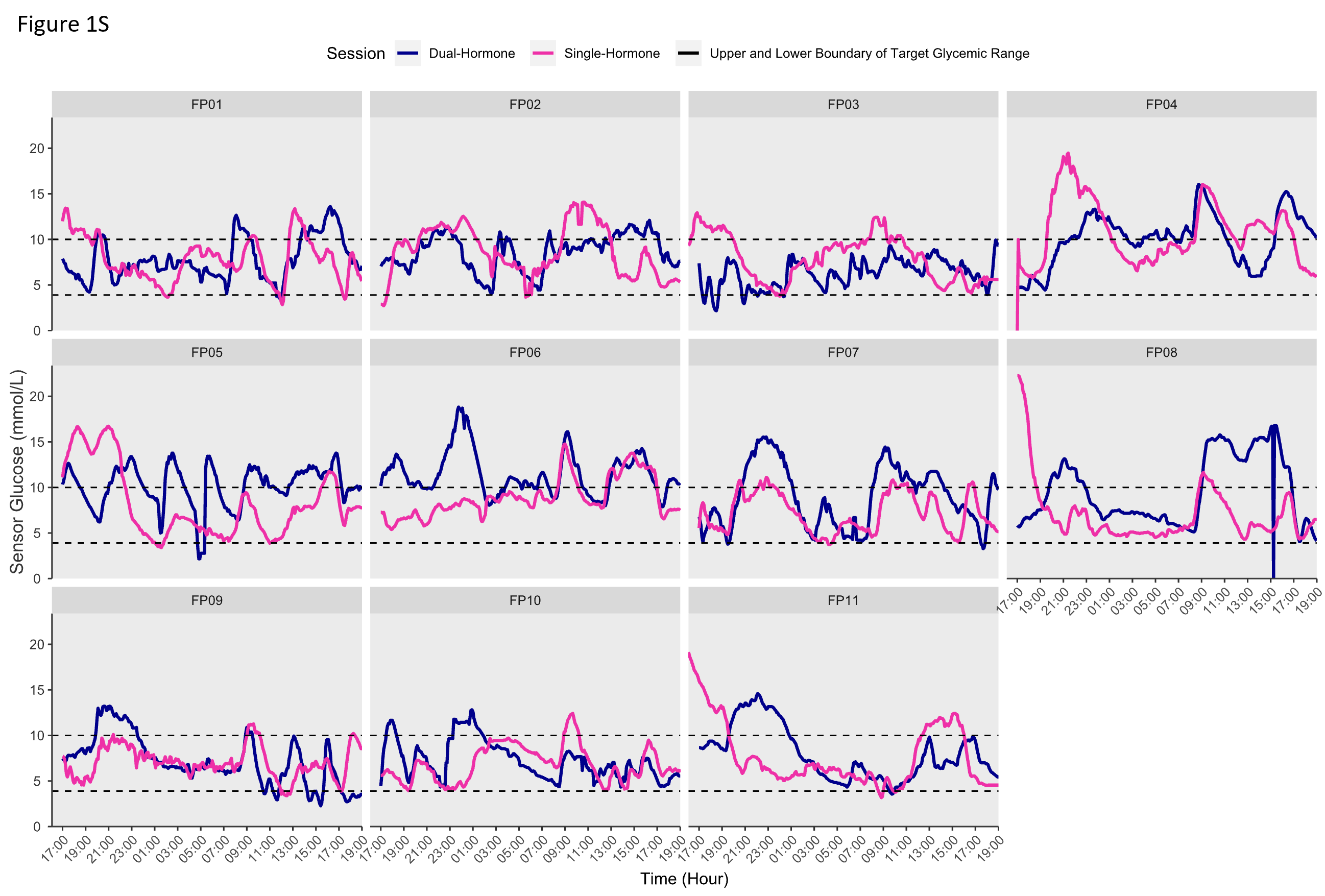

Supplement: Figure 1S: — Individual SG measures for participant during the entire study period for dual-hormone (blue) and single-hormone (pink). Black dotted lines mark the lower and upper boundary of target glycemic range of 3.9 mmol/L to 10.0 mmol/L, respectively. [file Image_1.tif]

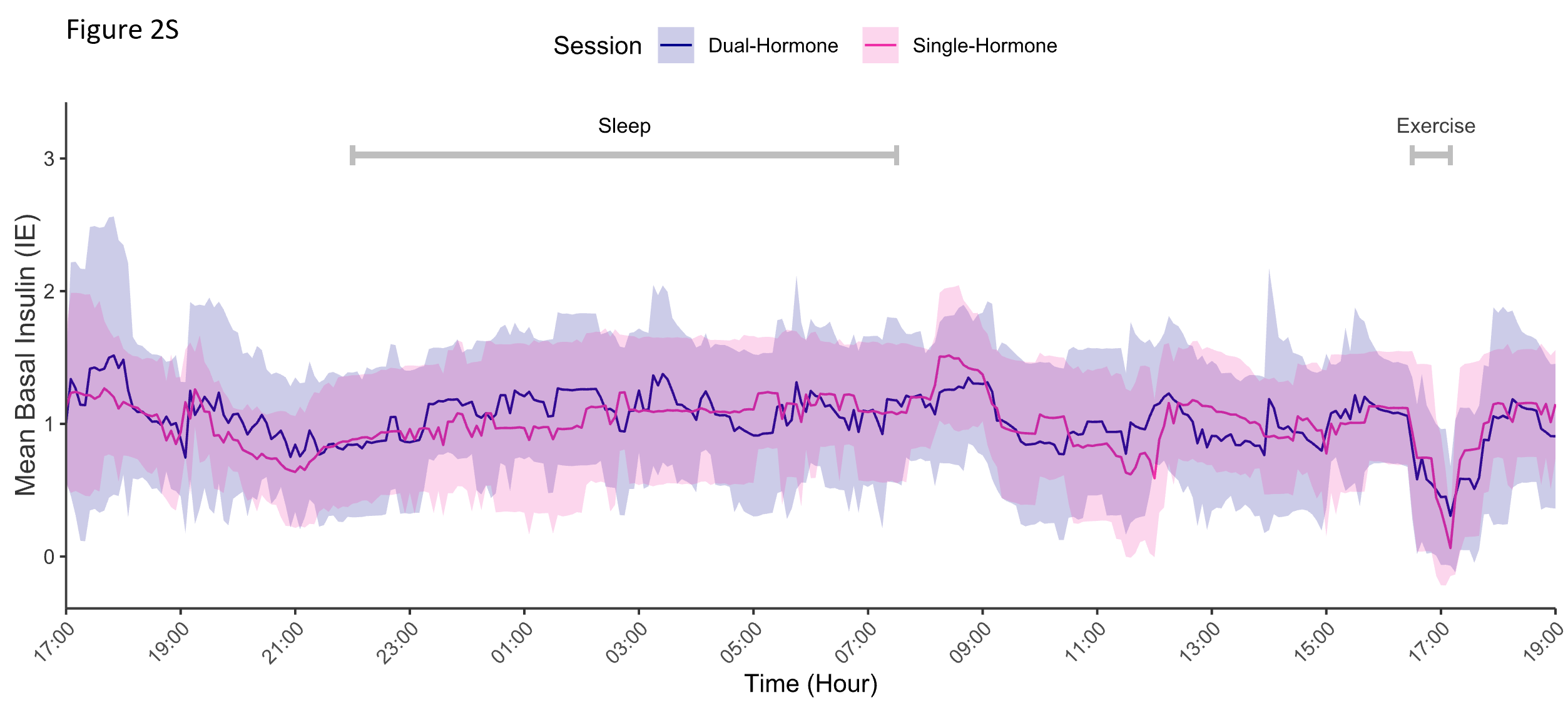

Supplement: Figure 2S: — Mean (±SD) basal insulin (IE) delivered during the entire study period for dual-hormone (blue) and single-hormone (pink). Sleep and exercise periods marked in the figure. [file Image_2.tif]

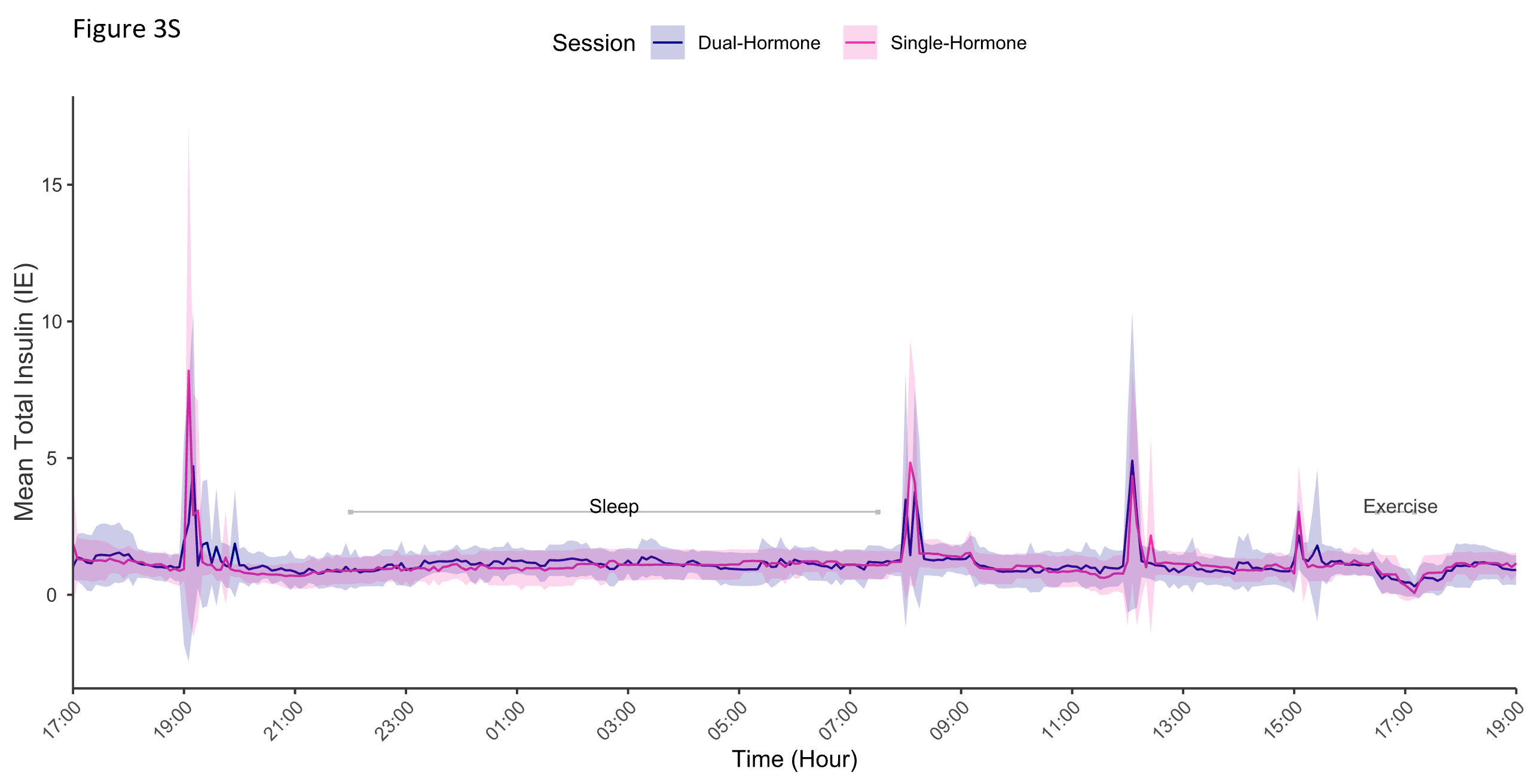

Supplement: Figure 3S: — Mean (±SD) total insulin (IE) delivered during the entire study period for dual-hormone (blue) and single-hormone (pink). Sleep and exercise periods marked in the figure. [file Image_3.tif]

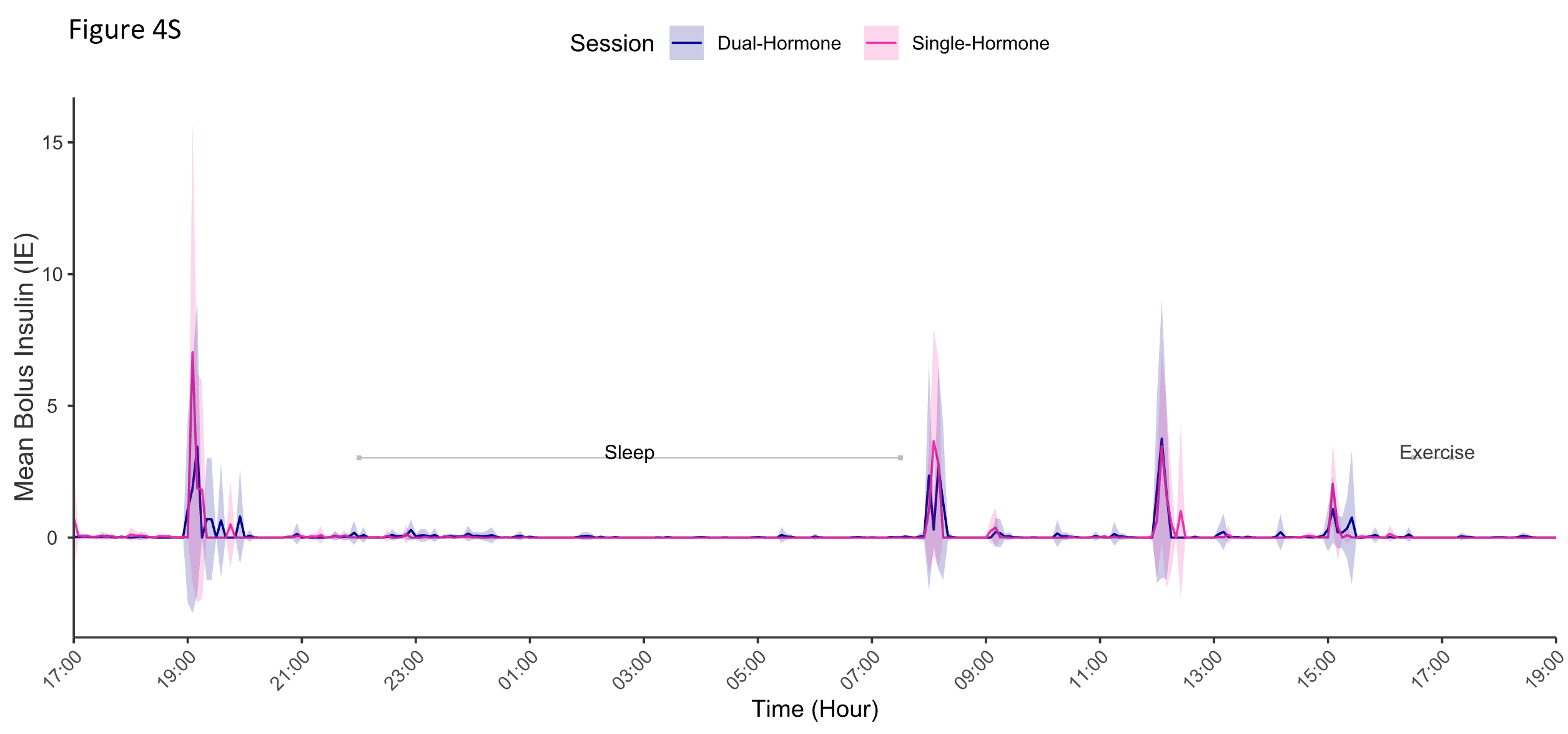

Supplement: Figure 4S: — Mean (±SD) bolus insulin (IE) delivered during the entire study period for dual-hormone (blue) and single-hormone (pink). Sleep and exercise periods marked in the figure. [file Image_4.tif]

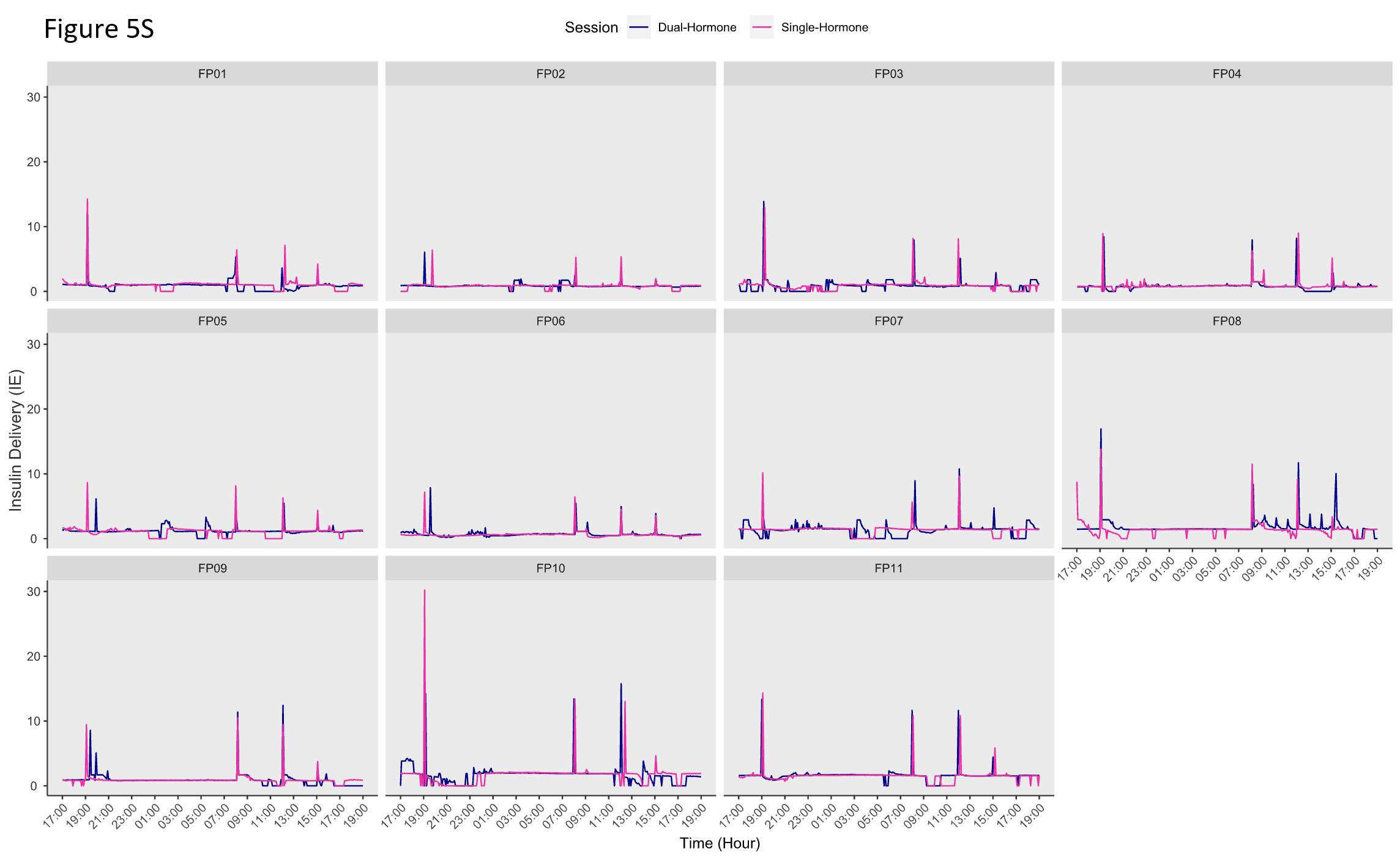

Supplement: Figure 5S: — Individual insulin delivery for each participant during the entire study period for dual-hormone (blue) and single-hormone (pink). [file Image_5.tif]

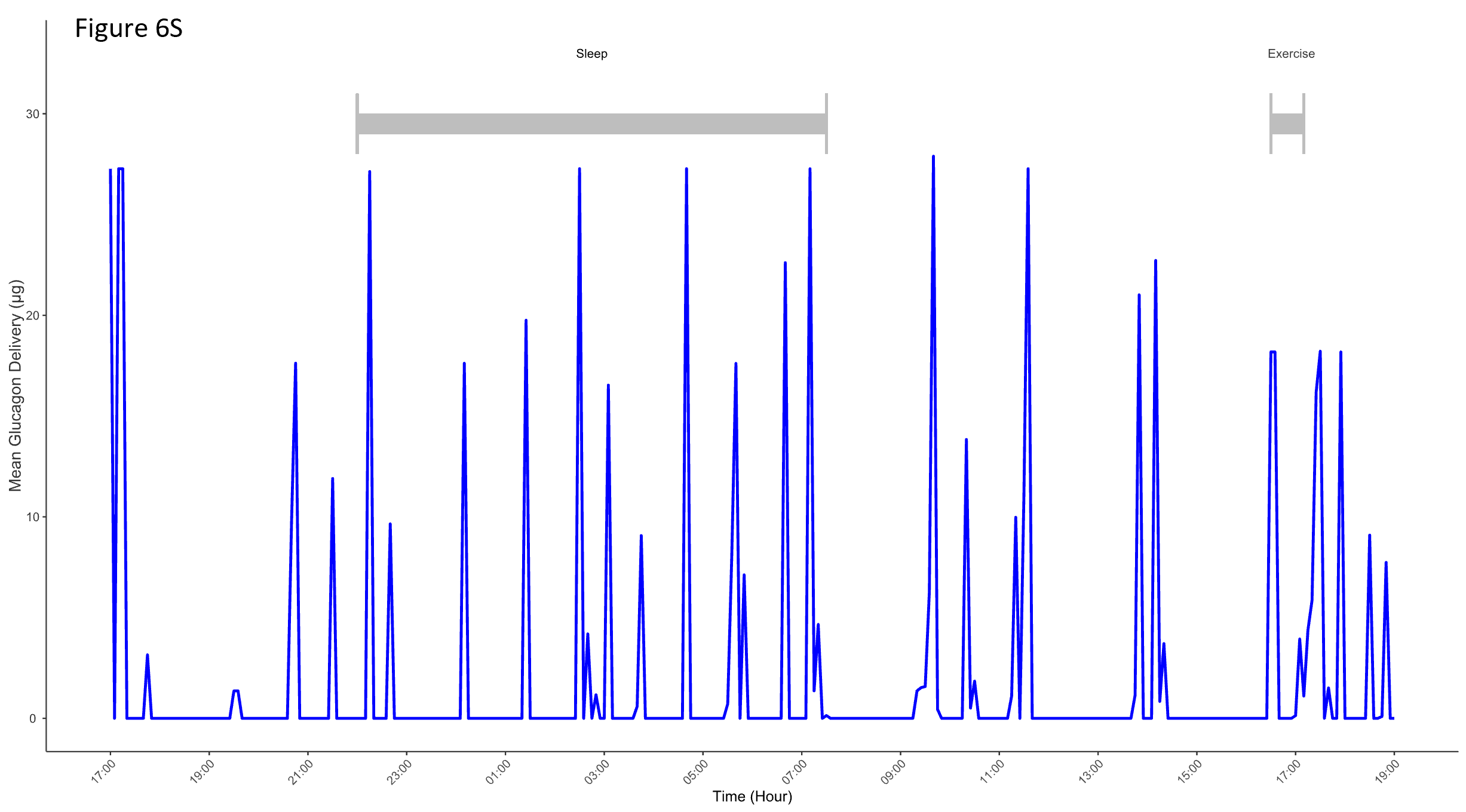

Supplement: Figure 6S: — Mean glucagon delivery during the entire dual-hormone study period. Sleep and exercise periods marked in the figure. [file Image_6.tif]

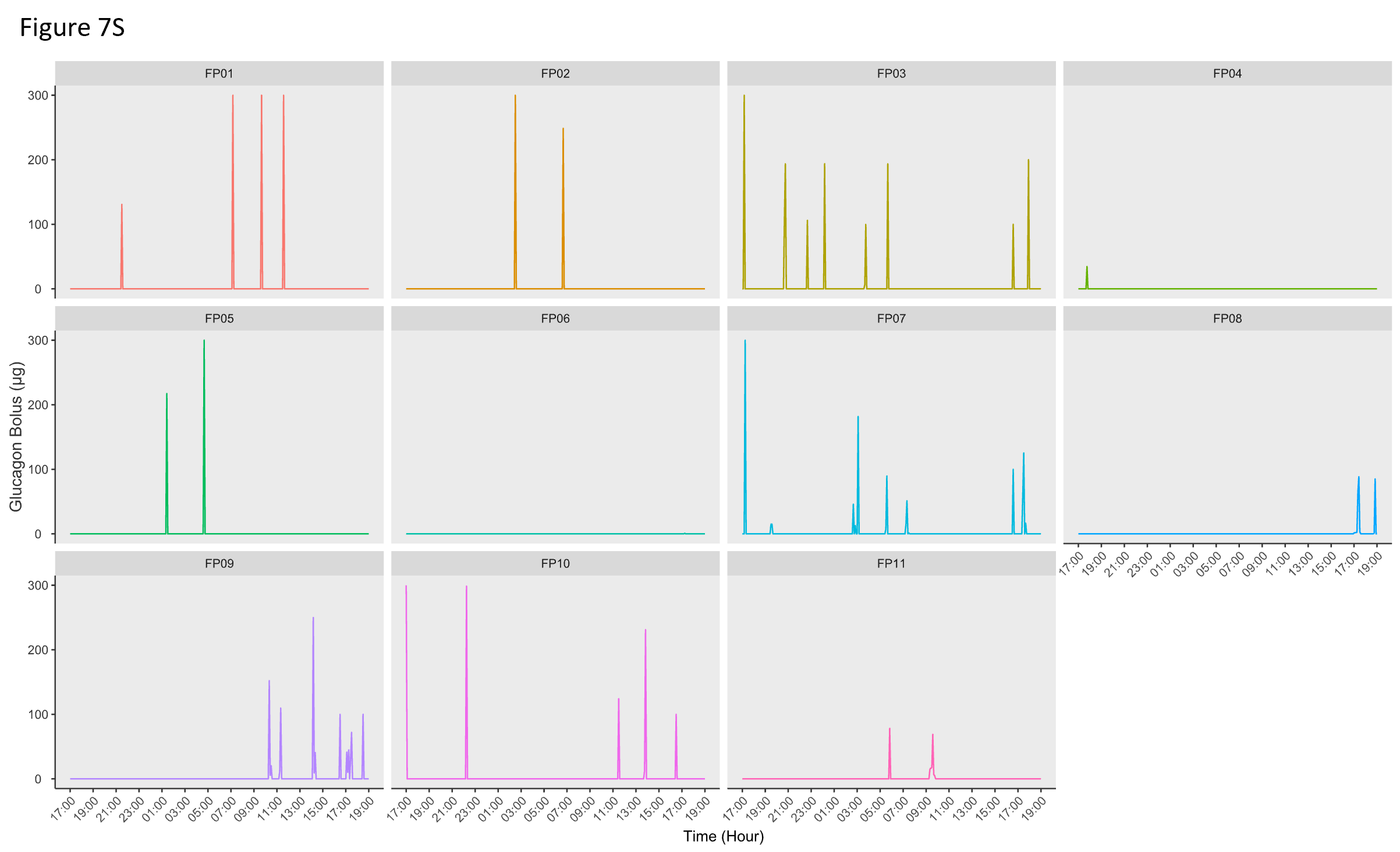

Supplement: Figure 7S: — Individual glucagon delivery for each participant during the entire dual-hormone study period. [file Image_7.tif]
